# Failure of bumetanide to improve outcome after intracerebral hemorrhage in rat

**DOI:** 10.1371/journal.pone.0210660

**Published:** 2019-01-10

**Authors:** Cassandra M. Wilkinson, Brittany A. Fedor, Jasmine R. Aziz, Colby A. Nadeau, Paul S. Brar, Julia J. A. Clark, Frederick Colbourne

**Affiliations:** 1 Department of Psychology, University of Alberta, Edmonton, Alberta, Canada; 2 Neuroscience and Mental Health Institute, University of Alberta, Edmonton, Alberta, Canada; Massachusetts General Hospital/Harvard Medical School, UNITED STATES

## Abstract

After intracerebral hemorrhage (ICH), brain edema commonly occurs and can cause death. Along with edema, there are significant alterations in the concentrations of key ions such as sodium, potassium, and chloride, which are essential to brain function. NKCC1, a cation-chloride cotransporter, is upregulated after brain damage, such as traumatic injury and ischemic stroke. NKCC1 brings sodium and chloride into the cell, possibly worsening ion dyshomeostasis. Bumetanide, a specific NKCC1 antagonist, blocks the transport of chloride into cells, and thus should attenuate the increases in chloride, which should lessen brain edema and improve neuronal functioning post-ICH, as with other injuries. We used the collagenase model of ICH to test whether bumetanide treatment for three days (vs. vehicle) would improve outcome. We gave bumetanide beginning at two hours or seven days post-ICH and measured behavioural outcome, edema, and brain ion content after treatment. There was some evidence for a minor reduction in edema after early dosing, but this did not improve behaviour or lessen injury. Contrary to our hypothesis, bumetanide did not normalize ion concentrations after late dosing. Bumetanide did not improve behavioural outcome or affect lesion volume. After ICH, bumetanide is safe to use in rats but does not improve functional outcome in the majority of animals.

## Introduction

Intracerebral hemorrhage (ICH) accounts for approximately 15% of all strokes and has a devastating 40% mortality rate [1]. Rehabilitation can improve outcome after ICH, but these improvements are limited, often leaving survivors with severe disabilities [2]. There is no current pharmacological intervention to mitigate injury or drive recovery after ICH, but there are many therapeutic targets, including cerebral edema and ion dyshomeostasis.

Cerebral edema, an increase in brain water content, commonly occurs in ICH patients and its resolution is thought to lead to behavioral recovery [3]. Edema is also seen in animal models, such as with collagenase infusion, and results from serum extrusion, blood-brain barrier (BBB) damage, and cell death, as well as water and ion transporter dysfunction, all of which alters intra- and extracellular ion concentrations [4,5]. This occurs quickly and persists for two or more weeks after ICH in rats [2,6]. For instance, BBB damage occurs after ICH due to both primary and secondary injury, thereby allowing for the free passage of ions and other molecules, including water [5,7]. While BBB damage contributes to cerebral edema and ion dyshomeostasis, it appears that the latter extends well beyond the time when BBB damage and edema resolves [2,5]. Using x-ray fluorescence imaging (XFI) we documented persistent changes in the collagenase model of ICH, and observed a reduction in potassium (K^+^) and an increase in total chloride (Cl^-^) concentrations at 14 days post-stroke [5]. For example, Cl^-^ was significantly increased from as far out as 900 μm into the peri-hematoma tissue. Na^+^ cannot be measured with XFI and was not assessed in that study. As edema typically resolves within the first week, we believe that additional mechanisms are likely involved, including key ionic transporter level alterations after ICH and perhaps a failure of hemostatic mechanisms to restore ionic balance. The Na^+^ Cl^-^ cotransporter NKCC1 is upregulated after brain damage and has been implicated in edema formation after both ischemia and traumatic brain injury; it is also believed to contribute to edema in hemorrhagic stroke [8–12]. Expression of NKCC1 on neurons and glial cells upregulates after ischemia and decreased ATP-ase activity [13,14]. Upregulation of NKCC1 leads to an influx of Na^+^ and Cl^-^ into cells, exacerbating ion dyshomeostasis. Other ionic receptors, including the Sur1-Trpm4 sulfonylurea receptor, are upregulated after ICH and may contribute to further ionic dyshomeostasis [15]. It is not known which receptors or combination of receptors are most important. Expression studies alone cannot conclusively determine a receptor’s contribution to ionic dyshomeostasis; even those receptors that do not change may still play an important role.

Disrupted ion gradients likely cause neuronal dysfunction after ICH, including seizures that occur in the collagenase model [16,17] as well as in patients [18]. Further, ICH patients experience disorganized and disrupted alpha and delta rhythms, related to the location of the hematoma [19]. Presumably, restoring ion homeostasis is beneficial, and recent work supports this idea. For instance, rehabilitation, which improves behavioural outcome, has been shown to partially normalize Cl^-^ and K^+^ concentrations after ICH [2]. In this study, we focused on the actions of the NKCC1 receptor, in an attempt to mimic the normalization of Cl^-^ levels that is observed after rehabilitation.

Bumetanide, a specific NKCC1 antagonist, is a clinically approved loop diuretic used to treat heart failure and reduce edema. Current clinical use and the drug’s ability to reduce edema make bumetanide a promising candidate to treat ischemic, hemorrhagic, and traumatic brain injury [20–23]. Bumetanide blocks influx of Na^+^ and Cl^-^, which are both present in excess after ICH. As such, bumetanide has been explored as a seizure treatment and has the potential to reduce ion dyshomeostasis after ICH [8,24,25]. For these reasons, the impact of bumetanide on ICH must be explored.

Here, we evaluated the effectiveness of bumetanide as a treatment for ICH in rats, produced by intra-striatal infusion of collagenase. This model causes more extensive BBB damage and more edema than the autologous whole blood infusion [4] and perhaps better reflects the amount of BBB disruption and edema experienced in patients [4,5,26]. Additionally, ion dyshomeostasis is present for at least two weeks after collagenase ICH, giving a large therapeutic window in which bumetanide could theoretically provide benefit [2]. In patients, there is also a large therapeutic window in which electrical imbalances can be addressed, with post-ICH seizures occurring from onset until months after the stroke [27,28]. It should be noted that seizures were not observed in the whole blood model of ICH [16], but they do occur in the collagenase model [16,29]. Initially, we assessed the impact of bumetanide given two hours post-ICH on edema and behaviour. We examined multiple dosing regimens, varying the amount, frequency, and route of administration. Next, we assessed whether bumetanide affects ion concentrations, behaviour, and lesion volume when given seven days post-ICH, a time when edema and BBB permeability largely resolves but ion dyshomeostasis is still present [2,5].

## Materials and methods

### Subjects

Procedures were in accordance with the Canadian Council on Animal Care Guidelines and were approved by the Biosciences Animal Care and Use Committee at the University of Alberta. All surgical procedures were performed under isoflurane anesthesia, and analgesics were used to minimize pain. Animals’ health and behaviour were monitored at least twice daily.

We used 147 male Sprague Dawley rats (250-500g, approximately 2–4 months) from Charles River (Saint Constant, Quebec). Water and Purina rodent chow were provided *ad libitum*, except during experiment 6 where food deprivation was required. Rats were single-housed, except in experiments 4 and 6, in which they were housed in groups of four. Rats were kept in a temperature- and humidity-controlled room with lights on from 7 am-7 pm.

**Fig 1:**
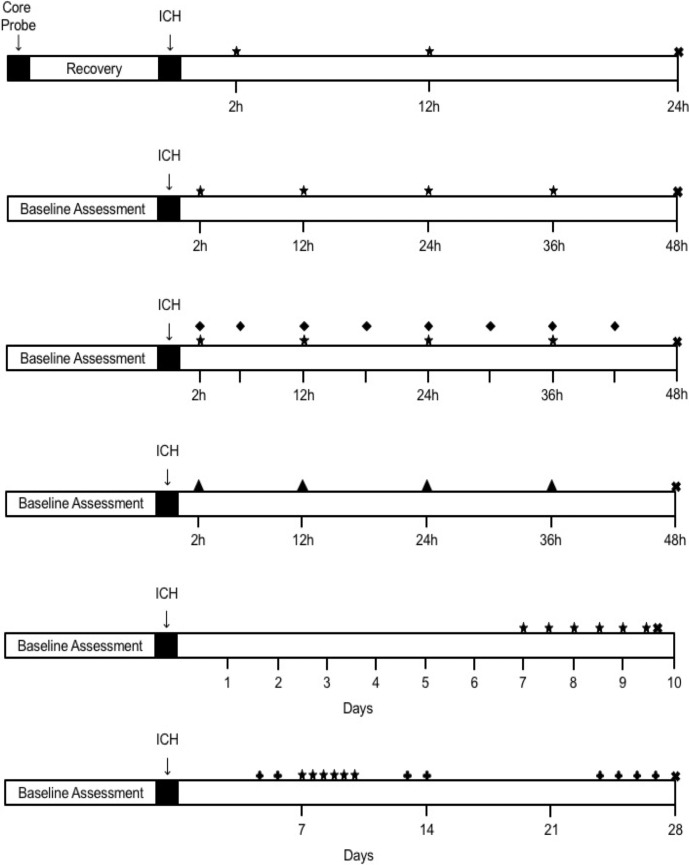
Experimental Timelines. (**A**) Experiment 1. (**B**) Experiment 2. (**C**) Experiment 3. (**D**) Experiment 4. (**E**) Experiment 5. (**F**) Experiment 6. ★ denotes oral dose administered on 12-hour interval. ♦ denotes oral dose administered on 6-hour interval. ▲ denotes intraperitoneal (IP) injection on 12-hour interval. ✚ denotes behaviour testing day. ✖ denotes euthanasia.

For all experiments, animals were randomized to group using random.org. All data collection and analysis were done by experimenters blinded to group assignment. Power analyses were based on the observed power for the primary endpoint of each experiment. For experimental timelines, see Fig 1.

In experiment 1, animals were randomized to 40 mg/kg bumetanide or vehicle groups (n = 8 each). This sample size gave 80% power to detect a 40% increase or decrease in hematoma volume. An additional 3 rats were used solely to assess the impact of bumetanide on blood pressure.

In experiment 2, animals were randomized to 40 mg/kg bumetanide or vehicle (n = 6 each). This sample size gave 80% power to detect a 1.5% change in brain water content.

Due to the short half-life of bumetanide, we assessed a more frequent dosing regimen in experiment 3. With this frequency, we looked at the effects of having bumetanide bioavailable for a larger proportion of time than only using two doses per day. Animals were randomized to 40 mg/kg bumetanide every 12 hours, 6 hours, or vehicle (n = 8 each). This sample size gave 85% power to detect a 1.5% change in edema. Regardless of group assignment, all animals received a dose of drug or vehicle every 6 hours to eliminate dosing confounds.

Due to the potential variability of oral dosing (e.g., consumption time between 1–10 minutes), we assessed intraperitoneal bumetanide injections in experiment 4. Injections were precisely timed. Animals were randomized to 40 mg/kg bumetanide or vehicle (n = 10 each). With this sample size, we had 92% power to detect a 1.5% change in edema.

In experiment 5, we assessed a delayed dosing regimen to a time when injury processes are starting to resolve and repair processes are engaged. In essence, we attempted to augment the recovery and repair processes by restoring the ionic environment. Animals were randomized to 40 mg/kg bumetanide or vehicle (n = 12 each). This sample size gave 85% power to detect at 20% change in iron (Fe) and 95% power to detect a 10% change in Na.

For experiment 6, each cage of four animals was randomized to 10 mg/kg bumetanide, 40 mg/kg bumetanide, or vehicle (n = 16 each). This sample gave 80% power to detect a 70% change in total lesion volume.

### Bumetanide administration

As bumetanide is readily absorbed orally [30], rats were given bumetanide mixed into 250 μL of canola oil and 250 mg of powdered cake mixture (Betty Crocker™) for experiments 1, 2, and 3. Our highest dose assessed was 40 mg/kg, which was the most likely dose to have potentially harmful side effects, as assessed in experiment 1. This dose is consistent with the doses others have used for ischemia and spinal cord injury [22,31]. Previous literature has used a large range of bumetanide doses [9,22,25,31], with 40 mg/kg representing the upper range of these. Doses were given by free-feeding, and rats readily consumed this mixture despite motor deficits. Oral dosing eliminated the stress response to injections. Vehicle treated rats were given equivalent amounts of canola oil and cake batter, and all animals were given this mixture prior to ICH surgery to ensure familiarity with the food. Cake mixture given prior to ICH did not include bumetanide. Animals were single housed for the duration of dose consumption in all oral dosing experiments. Refer to Fig 1 for a complete illustration of the dosing schedule in all experiments.

In experiment 4, bumetanide was dissolved in filter sterilized peanut oil and animals received intraperitoneal injections of bumetanide or oil alone. The dose was kept consistent with oral dosing experiments, as previous literature used a similar dose administered intraperitoneally [22].

For experiments 5 and 6, rats were given bumetanide mixed into three grams of sugar cookie dough (Pillsbury) or cookie dough alone. Coloured dough was used to verify that rats consumed the entire dose. Rats were given cookie dough prior to surgery to ensure consumption of serving. By seven days post-ICH, animals had recovered enough to readily consume cookie dough.

### Telemetry probe implantation

In experiment 1, animals were anaesthetized with isoflurane (4% induction, 2–2.5% maintenance, 60% N_2_0, and remainder O_2_). A midline incision was made in the abdomen and a sterile telemetry probe was inserted (Model TA10TA-F40, Data Sciences International, St. Paul, MN). These probes are accurate to ±0.1°C. Baseline temperature and activity was recorded every 30 seconds from 24 hours prior to ICH until euthanasia. Marcaine (0.5 mg S.C., Pfizer Canada) and Metacam (0.2 mg/kg S.C.) were administered for analgesia.

For blood pressure measurements, a calibrated PA-C10 probe’s catheter (Data Sciences International, St. Paul, MN; ± 3 mmHg accuracy) was inserted into the femoral artery while the probe was implanted subcutaneously, as previously described [32].

### Collagenase model

Animals were anaesthetized with isoflurane and temperature was maintained as previously described [33]. Rats were placed in a stereotaxic frame and a hole was drilled 0.5 mm anterior and 3.5 mm lateral from Bregma. Bacterial collagenase (Type IV-S, Sigma, 1.0 μL of 0.6 U/μL) was injected over 5 minutes via a 26 G needle lowered 6.5 mm in depth. The needle was left in place for 5 minutes to prevent backflow. The hole was sealed with a small metal screw and incision was stapled closed. Marcaine (0.5 mg S.C.) was applied for analgesia. Animals were given collagenase infusions in the left striatum, except in experiment 6, in which collagenase was infused contralateral to the dominant paw as determined by the skilled reaching assessment. Animals were randomized after surgery was complete.

### Hydration assessment

Due to the diuretic effects of bumetanide [34], we assessed water consumption and tissue hydration. Water bottles were measured to determine amount of water consumed. In experiment 4, water bottles were not measured due to mixed treatment group housing. In experiment 6, water bottles were measured per cage of four, as animals were group housed and randomized by cage. In this case, each data point in our analysis represents a cage of animals, not an individual rat. At the time of euthanasia, a cardiac blood sample was taken and allowed to clot for approximately 15 minutes. Blood was centrifuged for 30 minutes at 15.8g. Amount of serum in sample was expressed as percent serum and used as a measure of hydration. Samples of abdominal muscle and thigh muscle were taken immediately following euthanasia. Muscle water content was determined using the wet weight-dry weight method and used as an additional measure of hydration [35].

### Hematoma volume assay

Blood volume in the ipsilateral and contralateral hemispheres was measured using a spectrophotometric hemoglobin assay on homogenized samples, as described previously [2]. Rats were euthanized 24 hours post-ICH. Hematoma volume was expressed as ipsilateral hemisphere’s blood volume minus contralateral hemisphere’s blood volume. This was to control for blood in the vasculature.

### Neurological deficit scale

The neurological deficit scale (NDS) was graded based on the scoring of five subtests: spontaneous circling, beam walking, bilateral forelimb grasping, contralateral hind limb retraction, and contralateral forelimb flexion, as described previously [36]. Composite score ranged from 0–14, with 0 meaning no deficits and 14 meaning the greatest deficits.

### Brain water content

Brain water content was measured using the wet weight-dry weight method (baking at 100°C for 24 hr) at 48 hours post-ICH [35]. Tissue samples were taken 2 mm anterior until 4 mm posterior to the infusion site and separated into cortical and striatal tissue. The cerebellum was used as a control.

### Inductively-coupled plasma mass spectrometry (ICP-MS)

On day 9 post-ICH, brain tissue was dissected into striatal and cortical sections from 2 mm anterior to 4 mm posterior to the infusion site. Sections were digested in high purity nitric acid for one week and then assessed for Na, K, and Fe concentrations using ICP-MS (Thermo Scientific ICAP-Q quadrupole ICP-MS, Canadian Centre for Isotopic Microanalysis, University of Alberta) [5]. Cl concentrations cannot be measured with this method, and thus we used Na and K concentrations to measure ion dyshomeostasis. Fe concentration was assessed as a rough indicator of hematoma size when compared with the contralateral side.

### Skilled reaching

Animals were trained for four weeks (twice daily, five days per week) on the Montoya staircase task [36]. For training, animals were maintained at 90% of their free feeding weight. Animals were then tested on days 5–6, 13–14, and 24–27 post-ICH. Baseline score was calculated as the average number of successful reaches with the dominant paw on the last three days of training. Animals with less than nine out of 21 successful pellet (45 mg reward pellets, BioServe) retrievals on either side were excluded from behavioural analysis (exclusion criteria were decided prior to experimentation), but still underwent staircase testing on all days and were otherwise included in the experiment.

### Histology

On day 28 post-ICH, animals were administered sodium pentobarbital (100 mg/kg IP) and transcardially perfused with saline (0.9%), followed by 10% neutral buffered formalin. Brains were extracted and fixed in formaldehyde for at least 7 days, after which they were transferred to a 20% sucrose solution for cryoprotection.

40-μm coronal sections were taken every 200-μm and stained with cresyl violet for lesion volume analysis, as previously done [36]. Briefly, the total volume of brain injury was assessed as the volume of the contralesional hemisphere minus the volume of the ipsilesional hemisphere. This accounts for direct tissue damage and atrophy (e.g., cavity formation and ventriculomegaly).

### Statistical analysis

All data are expressed as mean ± 95% confidence interval, except NDS scores, which are presented as median ± interquartile range (IQR). Two-group comparisons were analyzed using t-tests, except NDS scores which were analyzed using a Mann-Whitney U-Test or Kruskal-Wallis test. Welch’s correction was used when variances were unequal between groups. ANOVA with Sidak’s or Tukey’s post hoc test was used when multiple groups were compared. The effect of bumetanide on blood pressure was assessed using a repeated measures ANOVA. Outliers were identified using the extreme studentized deviant method. Chi-squared test was used to assess exclusion differences among groups. Shapiro-Wilk was used to assess normality. Data were analyzed using GraphPad Prism (version 6) or SPSS. A P-value of <0.05 was considered statistically significant.

Data from experiments 2–4 were pooled to look for very small effects that we did not have the power to detect in individual experiments. Although dosing regimens varied slightly between experiments, dosing timing and duration was consistent. Further, as this analysis was unplanned and exploratory in nature, it is not considered conclusive.

## Results

### Mortality and exclusions

There was no mortality. In both experiment 4 and experiment 6, one cardiac blood sample was excluded due to experimenter error (both from animals in the 40 mg/kg group). In experiment 5, three individual water measurements were lost from the vehicle group due to water bottle leaks. One ICP-MS sample in experiment 5 was excluded as it was 4.72 standard deviations above the group mean, a statistical outlier (*P<*0.001), and assumed to be an error. In experiment 6, one animal from the high dose group and one animal from the vehicle group were excluded due to failure to reach training criteria. Twelve individual water measurements were lost in experiment 6 due to support staff error, but this was a random occurrence among groups (*P* = 0.448). In all cases, individual data points were excluded but animals were otherwise included.

### Bleeding, lesion volume, temperature, activity, and blood pressure

**Fig 2:**
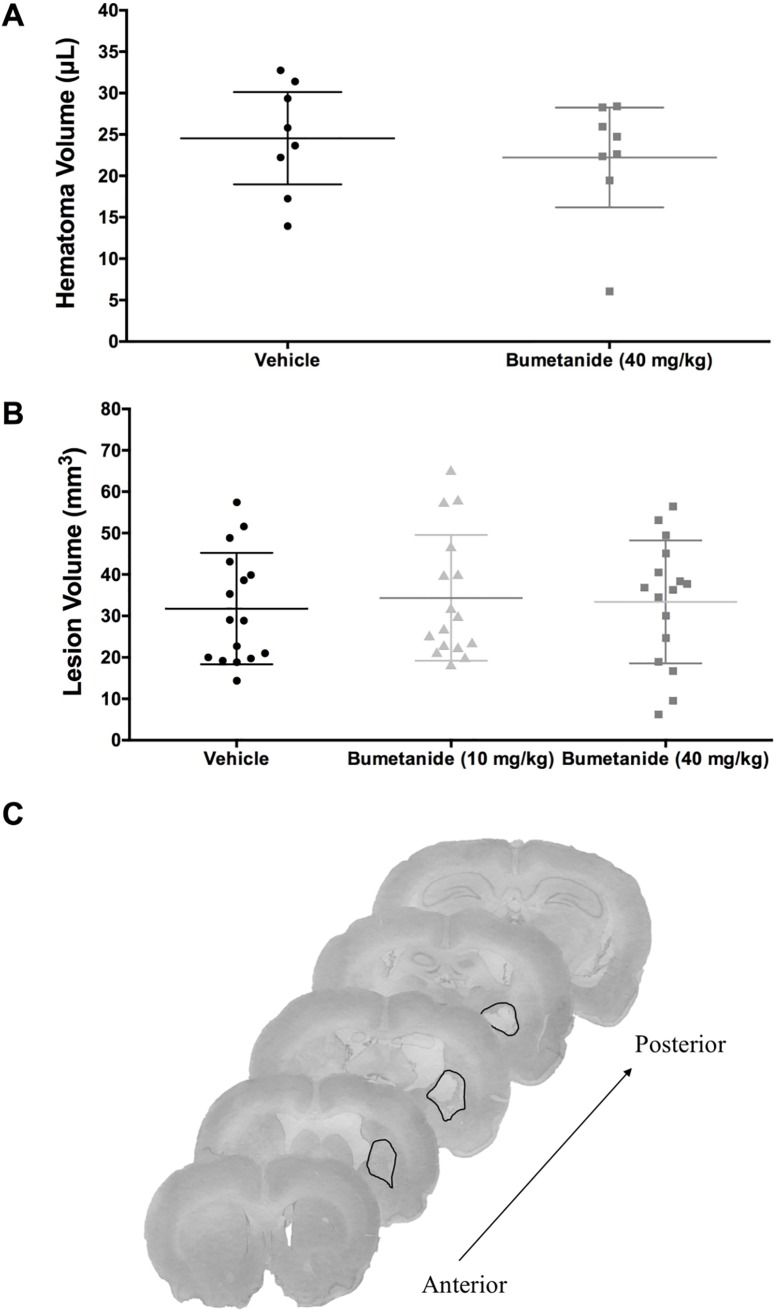
24-hour hematoma volume and 28-day lesion volume. (**A**) Hematoma volume (μL) data from experiment 1 (n = 8/group) showed that bumetanide did not significantly affect bleeding (*P* = 0.515). (**B**) Total tissue loss (mm^3^), including injury and atrophy, from experiment 6 (n = 16/group) showed that bumetanide did not significantly affect the volume of injury (*P* = 0.878). (**C**) Representative histological images at 28 days post-ICH. The black line demarcates the lesion border, and that along with atrophy (e.g., ventriculomegaly) is used to determine tissue loss. This rat, which was in the high dose group, had a total tissue loss of 49.44 mm^3^. All data presented as mean ± 95% confidence interval.

Bumetanide did not significantly affect bleeding at 24 hours post-ICH (Fig 2A, *P* = 0.515) or lesion volume at 28 days post-ICH (Fig 2B, *P* = 0.878).

**Fig 3:**
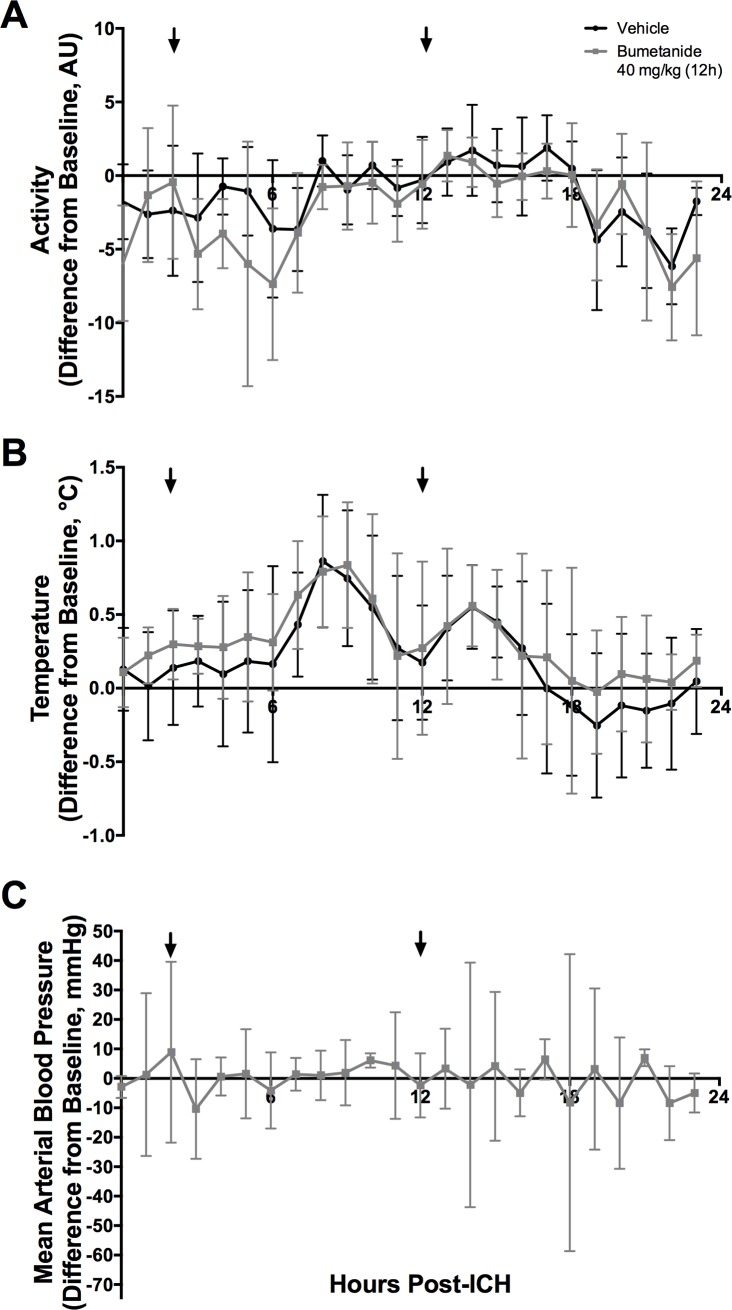
Temperature, activity, and blood pressure. (**A**) Activity (Arbitrary Units, AU) and (**B**) Temperature (°C) varied after ICH but were not significantly affected by bumetanide (*P*>0.10, group main effect; *P*>0.5, interaction effect, n = 8/group). All data presented as mean ± 95% confidence interval. (**C**) Blood pressure was not significantly affected by bumetanide (*P* = 0.83).

Temperature, activity, and blood pressure data were averaged hourly and normalized to the same hour at baseline to account for circadian rhythms. Temperature and activity varied over time after ICH (Fig 3, *P*<0.001) indicating a mild fever response that peaked 8 hours post-ICH. Neither temperature nor activity were impacted by bumetanide (Fig 3, *P*>0.100, group main effect; *P*>0.500, interaction effect). Bumetanide also did not affect blood pressure (Fig 3, *P* = 0.83).

### Brain water content

**Fig 4:**
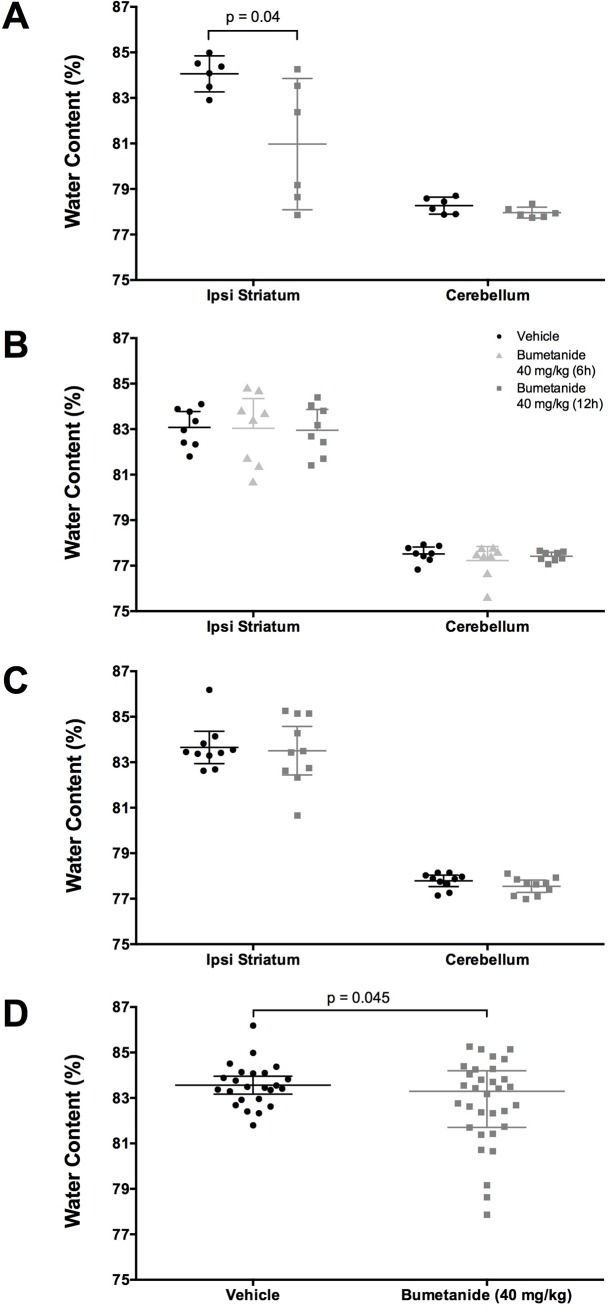
Brain water content. (**A**) Experiment 2 shows that bumetanide reduced brain water content (*P* = 0.040, n = 6/group). (**B**) In Experiment 3 (n = 8/group) and (**C**) Experiment 4 (n = 10/group) shows that bumetanide failed to reduce brain water content (*P* = 0.275 and *P* = 0.401 respectively). (**D**) Pooled brain water content of Experiments 2–4 shows that bumetanide significantly reduced edema (*P* = 0.045, n = 32 bumetanide-treated, n = 24 vehicle-treated). All data presented as mean ± 95% confidence interval.

In experiment 2, bumetanide reduced brain water content (Fig 4A, *P* = 0.040). However, in experiment 3 (Fig 4B, *P* = 0.275) and experiment 4 (Fig 4C, *P* = 0.401), bumetanide failed to reduce brain water content. When cerebral edema data from experiments 2, 3, and, 4 are analyzed together as an exploratory analysis, bumetanide was found to significantly reduce edema (Fig 4D, *P =* 0.045). This comparison has an observed power of 55%, and *d* = 0.527, statistically suggesting a medium effect size. However, bumetanide lowered water content by only 0.8%, which is unlikely to be biological significant (see behavioral findings).

### Ion concentrations

**Fig 5:**
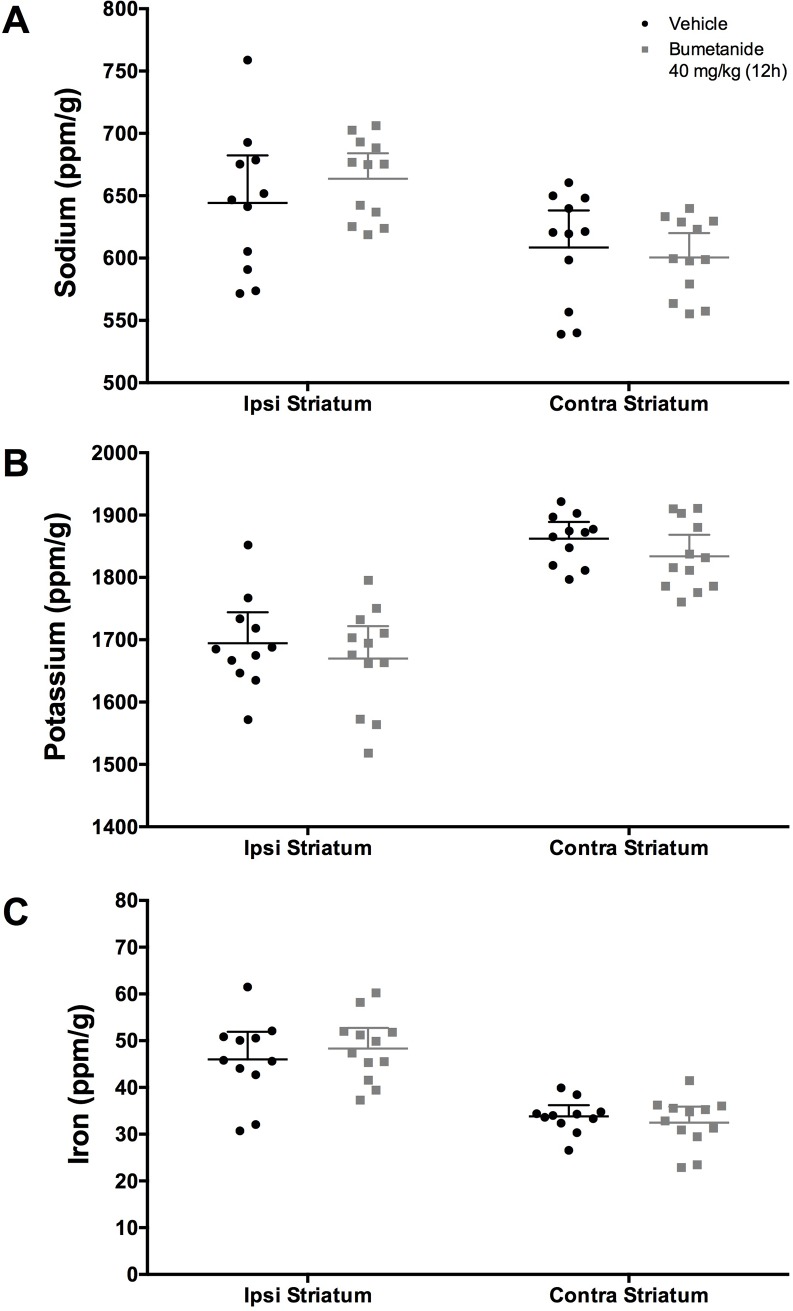
Ion concentrations in ipsilateral and contralateral striatum (ppm/g). Following ICH, (**A**) Na was increased (*P*<0.001), (**B**) K was decreased *P*<0.001), and (**C**) Fe was increased (*P*<0.001) in the ipsilateral striatum as compared to contralateral striatum (n = 11 bumetanide-treated, n = 12 vehicle-treated). Bumetanide did not significantly impact ion concentrations of Na, K, or Fe (*P≥* 0.125). All data presented as mean ± 95% confidence interval.

At 9 days post-ICH, Na was increased (Fig 5A, *P*<0.001, *d* = 1.199), K was decreased (Fig 5B, *P*<0.001, *d* = 2.578), and Fe was increased (Fig 5C, *P*<0.001, *d* = 2.181) in the ipsilateral striatum (vs. contralateral side). Bumetanide did not significantly impact Na, K, or Fe concentrations (Fig 5, *P*>0.125).

### Behaviour

**Fig 6:**
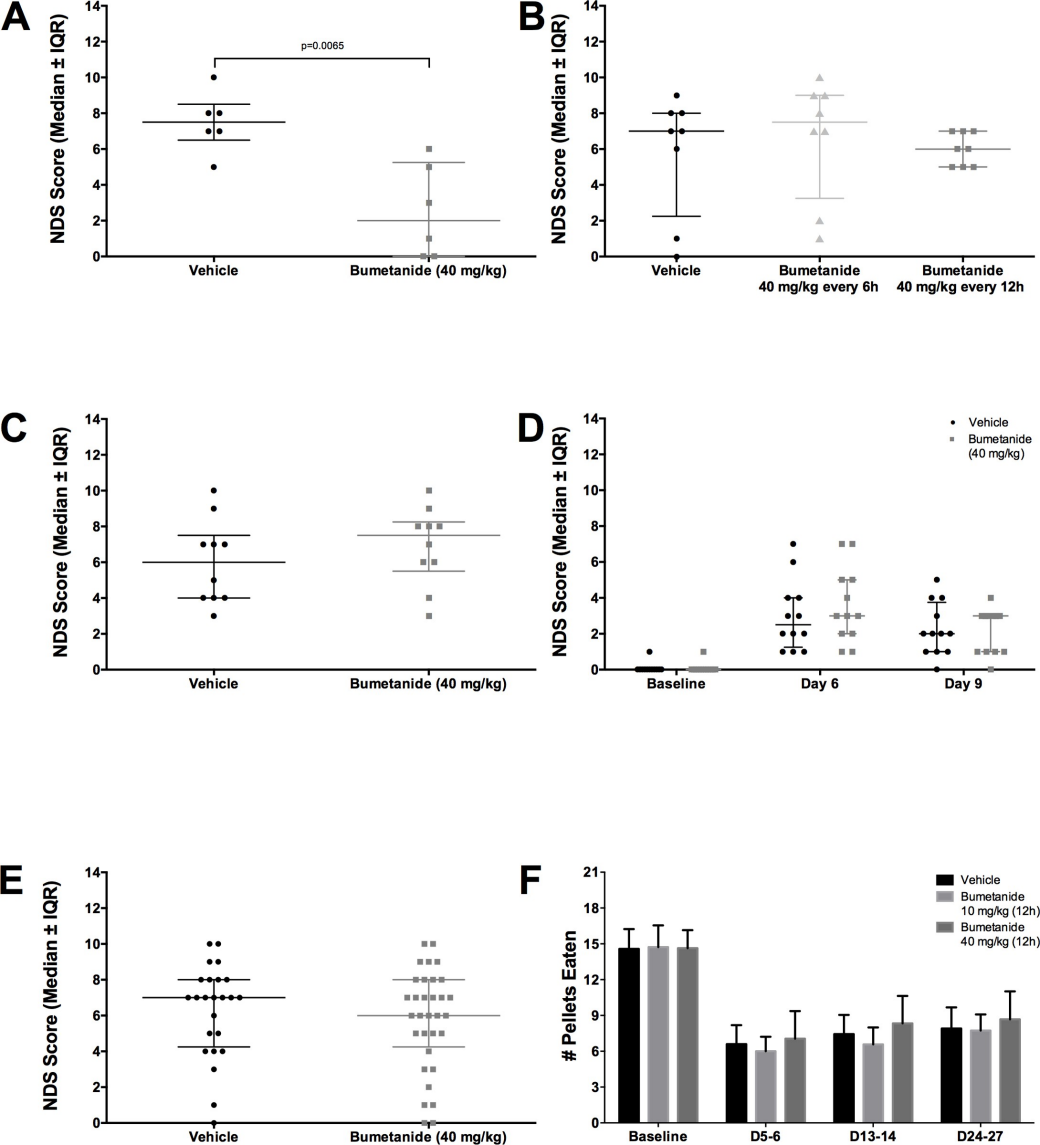
Behavioural assessment. **(A**) Data from Experiment 2 showed that bumetanide reduced NDS scores (*P* = 0.007, n = 6/group). This failed to replicate in (**B**) Experiments 3 (*P* = 0.295, n = 8/group), (**C**) 4 (*P* = 0.396, n = 10/group), and (**D**) 5 (*P* = 0.132, n = 12/group). (**E**) Pooled NDS Scores of Experiments 2–4 also failed to show an impact of bumetanide on neurological deficits *(P* = 0.488, n = 32 bumetanide-treated, n = 24 vehicle-treated). (**F**) Reaching success in Experiment 6 (n = 16/group) showed that ICH significantly reduced reaching ability (*P*<0.001), which bumetanide failed to improve (*P* = 0.587). (**A-E**) presented as median ± interquartile range, (**F**) presented as mean ± 95% confidence interval.

In experiment 2, bumetanide attenuated neurological deficits (Fig 6A, *P* = 0.007). Bumetanide failed to work in experiments 3 (Fig 6B, *P* = 0.295), 4 (Fig 6C, *P* = 0.396), and 5 (Fig 6D, *P* = 0.132). Pooling NDS scores from experiment 2, 3, and 4, as an exploratory analysis, shows that bumetanide did not significantly impact neurological outcome (Fig 6E, *P* = 0.488).

Reaching ability was significantly decreased post ICH (Fig 6F, *P*<0.001), but was unaffected by bumetanide (Fig 6F, *P* = 0.587, group main effect; *P* = 0.875, interaction effect).

### Hydration

**Fig 7:**
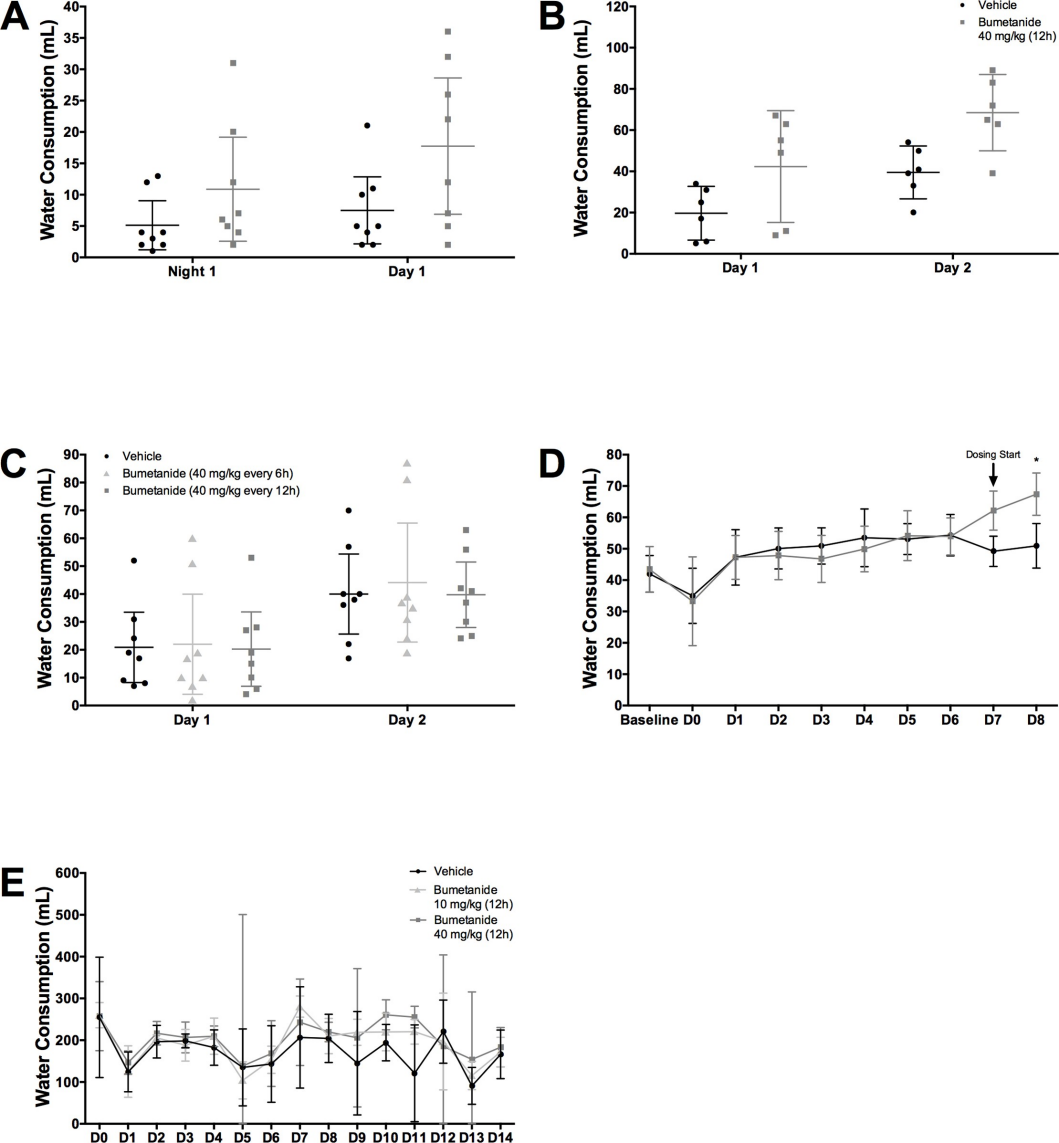
Hydration. Animals in the bumetanide groups drank significantly more water in (**A**) Experiments 1 (*P* = 0.034, group main effect, n = 8/group), (**B**) 2 (*P* = 0.023, group main effect, n = 6/group), (**D**) 5 (*P*<0.001 at Day 8, n = 12/group), (**E**) and 6 (*P =* 0.029, group main effect, n = 4 cages/group), but not in (**C**) Experiment 3 (*P* = 0.932, group main effect, n = 8/group). All data presented as mean ± 95% confidence interval.

Animals in the bumetanide group drank significantly more water in experiments 1 (Fig 7A, *P* = 0.034, group main effect; *P* = 0.465, interaction effect), 2 (Fig 7B, *P* = 0.023, group main effect; *P* = 0.283, interaction effect), 5 (Fig 7D, *P*<0.001 for Day 8; *P<*0.001, interaction effect), and 6 (Fig 7E, *P =* 0.029, group main effect; *P* = 0.917, interaction effect). Bumetanide did not impact water consumption in experiment 3 (Fig 7C, *P* = 0.932, group main effect; *P* = 0.999, interaction effect).

Bumetanide increased blood water content in experiment 4 (*P*<0.001), but not experiments 1 (*P* = 0.277), 2 (*P* = 0.942), 3 (*P* = 0.604), 5 (*P* = 0.583), or 6 (*P* = 0.537). Serum data are available in S1 Dataset.

Muscle water content was not affected by bumetanide in experiments 1 (*P* = 0.248, abdominal muscle; *P* = 0.587, thigh muscle), 2 (*P* = 0.246, abdominal muscle; *P* = 0.535, thigh muscle), 3 (*P* = 0.857, abdominal muscle; *P* = 0.640, thigh muscle), 4 (*P* = 0.789, abdominal muscle; *P* = 0.875, thigh muscle), or 6 (*P* = 0.634, abdominal muscle; *P* = 0.945, thigh muscle). In experiment 5, animals had significantly decreased abdominal (*P* = 0.027), but not thigh water content (*P* = 0.381). Muscle water content data are available in S1 Dataset.

## Discussion

Bumetanide, a selective NKCC1 inhibitor, can reduce edema in models of ischemia and traumatic brain injury at doses ranging from 15–30 mg/kg [20,31]. Others show that 30 mg/kg of bumetanide improves electrophysiological function as well as behaviour in a model of spinal cord injury, in a manner similar to rehabilitation [22]. In our ICH studies, bumetanide did not consistently reduce edema, although there was some indication that it may have modest effects. Bumetanide did not restore ion homeostasis, improve behavioural outcome, or lessen lesion volume. No benefit was seen despite using multiple dosing regimens, ranging from 10 mg/kg to 40 mg/kg, and multiple intervention times at two hours or seven days post-ICH. This is contrary to beneficial data seen in ischemia, traumatic brain injury, spinal cord injury, and epilepsy, with comparable dosing [20,22,23,31,37].

Although ICH shares similarities with ischemic and traumatic brain injury, there are important mechanistic differences. For example, the BBB is significantly damaged for weeks after ICH [5], likely longer than many other insults. This is partially due to mass effect, serum extrusion, and ongoing chronic injury from hematoma degradation, which do not occur in uncomplicated ischemia [38]. Within the first few days after collagenase ICH, the BBB is extensively damaged and is at peak permeability [4,5]. Despite inhibition of NKCC1 limiting Na+ and Cl- influx, these ions can still freely pass through the damaged BBB. Therefore, due to the extensive injury processes, bumetanide alone may not be enough to reduce edema and restore ion homeostasis in most animals when given acutely after ICH. As such, we delayed administration to a later time when injury processes, including BBB leakiness, are resolving, but this too had no impact. Combination therapies, such as blocking multiple receptors and/or mitigating BBB damage while targeting the NKCC1 receptor, may be required to treat edema and ionic dyshomeostasis in the acute and subacute periods after ICH [12].

Here, we replicate previous findings that Na is increased and K is decreased following ICH, and we add that ion dyshomeostasis is still present on day 9 post-ICH [2,5]. Bumetanide did not have an impact on ion concentrations, contrary to our expectations. Although ICP-MS does not provide spatial information, it is a precise and simple alternative to XFI, which has been used in previous studies [5,39,40]. One consideration when interpreting both ICP-MS and XFI data is the lack of delineation between intracellular and extracellular ion concentration. It is possible that the changes in ions are equivalent intra- and extra-cellular, and concentration gradients are preserved. The changes in electrical activity after collagenase infusion (e.g., seizures) would suggest alterations to the concentration gradients, but we cannot confirm this using ICP-MS or XFI [16]. Future studies using alternative methods, such as electrophysiology, are needed to determine the impact of ion dyshomeostasis.

Clinically, bumetanide is used to lower blood pressure and treat heart failure, specifically in patients who respond poorly to other loop diuretics [41–43]. Here, bleeding was not affected by bumetanide administration, which is not surprising given that blood pressure was not altered in our normotensive rats. The results of the current study suggest that it is safe to use or continue to use bumetanide after ICH, although further work would be needed to confirm this. Bumetanide did not exacerbate bleeding or negatively impact temperature or activity. Importantly, bumetanide did not worsen edema, behavioural outcomes, or lesion volume. Blood pressure is commonly elevated in ICH patients [43,44], and the above suggests that patients requiring a loop diuretic after ICH are unlikely to have brain injury worsened when using bumetanide.

The effects of bumetanide on water consumption indicate the drug was physiologically active. In the majority of experiments, animals in the bumetanide group consumed more water but did not have significantly altered blood serum or muscle water content. This suggests that bumetanide was exerting diuretic effects, which animals compensated for by drinking more water. In all but one experiment, rats in the bumetanide group drank significantly more water. We cannot be certain why water consumption was not affected by bumetanide in experiment 3, but a type two error is a possibility. With power kept at ~80% and a type two error rate maintained around 20%, we would certainly expect one out of five experiments to represent a false negative.

As often with negative studies, one can postulate that other dosage regimens might work in other models or insult severities, and so on. We cannot reasonably exclude such possibilities. However, we based the bumetanide dose on previous literature that found benefit at similar doses [22,31]. Further, we varied the frequency, magnitude, route, and timing of dosing to no avail. Rats quickly consumed the drug dose (within 30 min but often within a few min) and bumetanide is readily absorbed orally [30], which is the route of administration commonly used in patients taking the diuretic [45]. Therefore, it is unlikely that bumetanide failed to provide benefit based on the dose or route of administration. It is also possible that our results are a false negative. However, our sample sizes had adequate power to detect moderate effects in our primary endpoints. Further, it is not uncommon for results to fail to replicate or transfer to other insult types [46,47]. One factor making replication difficult is underpowered research overestimating effect sizes. Another factor is the overt positive publication bias, specifically in animal stroke research. In a previous review, only 2% of preclinical stroke studies were classified as negative [48], and as such it is more likely that this reflects publication bias than assuming virtually all therapies that were tested provided meaningful benefit (positive findings). This publication bias makes it very difficult to estimate true effect sizes and contributes to difficulty replicating and extending findings.

There are several limitations in the present study. First, bumetanide has poor brain penetration, which is perhaps not a problem in ICH owing to increased BBB permeability [49]. However, there is variability in BBB damage, which might contribute to group variability in treatment efficacy. Further, by seven days post-ICH, BBB damage has started to resolve [5]. This may have limited the availability of bumetanide to neuronal cells in experiment 5 and 6. We did not quantify the amount of bumetanide in the brain, but previous data shows bumetanide passes through an intact BBB in modest amounts [49,50]. Second, bumetanide may have had positive effects that were missed. For example, bumetanide can reduce seizure activity that occurs in the collagenase model [16]. Third, bumetanide has a short half-life of approximately two hours in rodents [30]. Therefore, maximum benefit may be achieved through continuous infusion of bumetanide. However, this is technically difficult as bumetanide is not readily soluble. Further, we saw no difference in efficacy between six and twelve-hour dosing, suggesting that increased dosing frequency may not provide additional benefit. Fourth, we did not directly assess NKCC1 receptor changes after ICH. The upregulation of NKCC1 in other forms of brain injury such as traumatic brain injury and ischemia [8–12] certainly suggest that NKCC1 would be upregulated post-ICH, but we did not confirm this. Further, the activity of NKCC1 has many factors, including receptor expression, intracellular cyclic adenosine monophosphate concentration, and calcium concentration [51,52]. Examining upregulation of NCKK1 alone would not conclusively demonstrate increased receptor activity. Regardless of the alterations in expression of NKCC1, bumetanide would still be able to act on the receptor as long as it was present in some concentration, such as in the substantial peri-hematoma region that have altered Na^+^, K^+^ and Cl^-^ levels. Finally, the effects of bumetanide may vary with model, age, sex, and comorbidities (e.g., spontaneous hypertension and diabetes).

In experiment 2, a pilot study, bumetanide reduced edema and neurological deficits. We were unable to replicate this finding in any of the following experiments. It is important to acknowledge the power in this experiment. Although we had sufficient power to detect a moderate effect (1.5% difference in edema), the true effect size of bumetanide may be quite small. There is a possibility that these results are what is known as the “Winner’s Curse,” and that our small sample overestimates the true effect size [46]. Ultimately, we cannot rely on the results of this experiment alone. When all of the edema results are pooled, it suggests bumetanide may have a modest effect on edema in some animals. The skewed nature of the data certainly shows that a subset of animals, but not all animals, see reductions in cerebral edema when administered bumetanide, and this reduction did not lead to improvements in neurological function. For example, eight bumetanide treated rats (of 32) had less edema than the lowest vehicle treated animal. Using this measure, we can conservatively estimate that 25% of animals might obtain modest reductions in edema from bumetanide administration. It is important to note that this pooled analysis was unplanned and exploratory in nature. Therefore, further work should be done to assess which animals benefit from bumetanide treatment and why such discrepancies exist in this population.

In conclusion, we have demonstrated that bumetanide is safe, but not beneficial, after ICH. Bumetanide may reduce cerebral edema modestly in a subset of the population. Treatment with bumetanide does not aggravate bleeding, worsen edema, or impact lesion volume. These findings suggest that despite benefit seen in other modes of brain injury, bumetanide does not significantly reduce edema or provide benefit after experimental ICH.

## Supporting information

S1 Dataset All data contained in this study. (XLSX)
